# Risk and protective genetic variants in suicidal behaviour: association with *SLC1A2, SLC1A3, 5-HTR1B *&*NTRK2 *polymorphisms

**DOI:** 10.1186/1744-9081-7-22

**Published:** 2011-06-28

**Authors:** Therese M Murphy, Maria Ryan, Tom Foster, Chris Kelly, Roy McClelland, John O'Grady, Eleanor Corcoran, John Brady, Michael Reilly, Anne Jeffers, Katherine Brown, Anne Maher, Noreen Bannan, Alison Casement, Dermot Lynch, Sharon Bolger, Prerna Tewari, Avril Buckley, Leah Quinlivan, Leslie Daly, Cecily Kelleher, Kevin M Malone

**Affiliations:** 1 Department of Psychiatry & Mental Health Research, St. Vincent's University Hospital, and School of Medicine & Medical Science, University College Dublin, Elm Park, Dublin 4, Ireland; 2 Tyrone & Fermanagh Hospital, Western Health and Social Care Trust, Omagh, Northern Ireland; 3 Department of Psychiatry, Queens University Belfast, Northern Ireland; 4 Department of Psychiatry, Letterkenny General Hospital, Letterkenny, Co. Donegal, Ireland; 5 Community Rehabilitation and Recovery Service, Primary Care Centre, Barrack Street, Sligo, Ireland; 6 St. Brigids Hospital, Ballinasloe, Co. Galway, Ireland; 7 St Fintans Hospital, Portlaoise, Co Laois, Ireland; 8 Department of Liaison Psychiatry, Connolly Hospital, Blanchardstown, Dublin 15, Ireland; 9 Institute of Molecular Medicine, Trinity Health Centre, St. James Hospital, Dublin, Ireland; 10 Clinical Research Centre, St. Vincent's University Hospital, and School of Medicine & Medical Science, University College Dublin, Elm Park, Dublin 4, Ireland; 11 Centre for Support and Training in Analysis and Research, University College Dublin, Belfield, Dublin 4, Ireland; 12 School of Public Health, Physiotherapy and Population Science, University College Dublin, Belfield, Dublin 4, Ireland

## Abstract

**Background:**

Suicidal behaviour is known to aggregate in families. Patients with psychiatric disorders are at higher risk for suicide attempts (SA), however protective and risk genetic variants for suicide appear to be independent of underlying psychiatric disorders. Here we investigate genetic variants in genes important for neurobiological pathways linked to suicidal behaviour and/or associated endophenotypes, for association with SA among patients with co-existing psychiatric illness. Selected gene-gene and gene-environment interactions were also tested.

**Methods:**

DNA was obtained from bloods of 159 patients (76 suicide attempters and 83 non-attempters), who were profiled for DSM-IV Axis I psychiatric diagnosis. Twenty-eight single nucleotide polymorphisms (SNPs) from 18 candidate genes (*COMT, 5-HT2A, 5-HT1A, 5-HTR1B, TPH1, MAO-A, TPH2, DBH, CNR1, BDNF, ABCG1, GABRA5, GABRG2, GABRB2, SLC1A2, SLC1A3, NTRK2, CRHR1*) were genotyped. Genotyping was performed by KBioscience. Tests of association between genetic variants and SA were conducted using Chi squared and Armitage Trend tests. Binary logistical regression analyses were performed to evaluate the contribution of individual genetic variants to the prediction of SA, and to examine SNPs for potential gene-gene and gene-environment interactions.

**Results:**

Our analysis identified 4 SNPs (rs4755404, rs2269272, rs6296 and rs1659400), which showed evidence of association with SA compared to a non-attempter control group. We provide evidence of a 3-locus gene-gene interaction, and a putative gene-environment interaction, whereby genetic variation at the *NTRK2 *locus may moderate the risk associated with history of childhood abuse.

**Conclusion:**

Preliminary findings suggest that allelic variability in *SLC1A2/3, 5-HTR1B *and *NTRK2 *may be relevant to the underlying diathesis for suicidal acts.

## Background

Suicide represents a worldwide public health problem and is a leading cause of death among those aged 15-44 years in many developed countries, representing a significant social and economic burden http://www.who.int/mental_health/prevention/suicide. According to the stress-diathesis model of suicidal behaviour, risk of suicide is not exclusively determined by a psychiatric illness (or an alternative stressor), but rather that individuals may have a predisposition or tendency towards suicidal ideation, which is aggravated further by one or more stressors [1]. A greater understanding of clinical and biological risk factors will help us understand more fully this model of suicidal behaviour and inform new therapies and tailored interventions.

The molecular pathology of suicidal behaviour remains complex; epidemiological studies have demonstrated familial clustering of suicide and suicidal behaviour [2]. Meta-analysis of published twin case studies for suicide found a higher rate of concordance for suicide and for suicidal behaviour in monozygotic (MZ) versus dizygotic (DZ) twins, establishing a solid genetic link [3]. Currently, risk assessment of suicidal behaviour relies heavily on clinical variables [4]. Indeed, patients with psychiatric disorders are at higher risk for suicide attempt (SA) [5-8], however protective and risk genetic variants for suicide appear to be independent of underlying psychiatric disorders [9-11]. Identifying relevant genetic variants or single nucleotide polymorphisms (SNPs) in genes intimately linked to various neurobiological pathways, whose alteration may contribute to suicidal behaviour, may provide insight into the etiological processes that underlie suicidal behaviour and identify novel biological therapeutic targets.

Numerous neurobiological pathways have been implicated in the etiology of suicide and suicidal behaviour. Deregulation of the monoaminergic transmitter systems (norepinephrine, dopamine and serotonin) [12,13], gamma aminobutyric acid (GABA) inhibitory neurotransmitter system [14,15], glutamatergic excitatory neurotransmitter system [14,15], lipid metabolism [16,17], cannabinoid system [18] and neurotrophic signalling factors [19] are linked to suicidal behaviour. Interestingly, the hypothalamic-pituitary-adrenal (HPA) axis may be involved in increased suicidal behaviour in response to adversity during development [20], hence highlighting the contribution of early environmental factors to suicide risk.

Suicidal behaviour is a complex trait; therefore it is likely to be the result of a combination of numerous environmental and genetic factors with an added layer of complexity via potential gene-gene (GxG) and gene-environment (GxE) interaction effects [21]. Consequently, investigating the relationship between genetic variants and environmental factors (e.g. childhood abuse or alcoholism) is an important step towards elucidating the mechanisms behind suicidal behaviour.

Here we test the hypothesis that genetic variants in candidate genes important for neurobiological pathways linked to suicidal behaviour and/or associated endophenotypes, may confer a protective [22] or risk effect on SA among patients with co-existing psychiatric illness (suicide attempters versus non-attempter controls). In addition to main genetic effects, we also tested selected GxG and GxE interactions for association with SA.

## Methods

### Clinical sample collection

The study consisted of 159 psychiatric patients recruited as part of the Ireland North/South Urban/Rural Epidemiologic (INSURE) collaborative project [23]. Briefly, consecutive newly referred patients to six Community Psychiatric Clinics on the island of Ireland (Donegal, Belfast, Omagh, Dublin, Portlaoise and Ballinasloe) were invited to take part in a clinical and molecular genetics study of suicidal behaviour in major psychiatric disorders over a year long recruitment period. In total, 159 patients gave written informed consent for the study. Patients were interviewed for Axis I Psychiatric Disorders using the Structured Clinical Interview for DSM-IV [24]. History of attempted suicide was recorded using the Columbia University Suicide History Questionnaire [25], which incorporates the Scale for Suicide Ideation [26], and the Suicide Intent Scale [27]. A suicide attempt was defined as a completed act of self-harm with at least some expressed intent to die (scoring at least 1 on item 10 of Suicide Intent Scale [27]). Seventy-six (48% Male, 52% Female; Mean age: 34.7 yrs) had a history of SA and 83 (61.4% Male, 38.6% Female; Mean age: 40.7 yrs) had not. Childhood abuse was defined as the presence or absence of a history of sexual and/or physical abuse under the age of 16. Further clinical variables from clinical and demographic assessments are outlined in Table 1. All clinical research interviews were performed by a trained research psychiatrist or research psychiatric nurse. Ethics approval was obtained for the study from the St Vincent's Healthcare Group Ethics and Medical Research Committee.

**Table 1:** DSM-IV psychiatric diagnosis and clinical features of patient samples

	Attempter (n = 76) n (%*)	Non-attempter (n = 83) n (%*)	P Value
**Sex**			0.124
Male	36(48)	51 (61.4)	
Female	39 (52)	32 (38.6)	
**Mean age (years)**	34.7	40.7	0.017
**Positive 1st degree family history of suicide attempt**	26 (35.6)	14 (17.1)	0.014
**Child abuse (Physical and/or sexual)**	37 (49.3)	27 (32.5)	0.047
**Axis I Diagnosis**	42 (55.3)	36 (43.4)	0.18
**Substance-related disorders**	24 (31.6)	21 (25.3)	0.334
Substance abuse/dependence	38 (50)	29 (34.9)	0.078
Alcohol abuse/dependence	6 (8.8)	7 (8.6)	1
**Schizophrenia and other psychotic disorders**			
**Bipolar disorders**	5 (7.4)	4 (4.8)	0.786
**Major depressive disorder**	48 (63.2)	37 (44.6)	0.022
**Anxiety disorders**	32 (42.7)	25 (30.1)	0.14
**Eating disorders**	1 (1.3)	0 (0)	0.965

§P value was determined by Fishers exact or Chi squared test in all comparisons, with the exception of age, where a non-parametric Mann-Whitney test was used to determine the P Value. *Illustrated percentages of variables are calculated for valid cases/controls only. In some cases certain clinical or demographic variables could not be obtained from patients charts.

### Gene and SNP selection

In this study, genes intimately linked to numerous neurobiological pathways considered important for the pathogenesis of suicide and/or associated disorders or endophenotypes, were selected. Genes from the following pathways were considered, monoaminergic transmitter systems (norepinephrine, dopamine and serotonin), GABA inhibitory neurotransmitter system, glutamatergic excitatory neurotransmitter system, HPA axis and cannabinoid system. Genes important for cholesterol transport and neural growth and differentiation were also selected.

Twenty-eight SNPs from the 18 selected genes (*COMT, 5-HT2A, 5-HT1A, 5-HTR1B, TPH1, MAO-A, TPH2, DBH, CNR1, BDNF, ABCG1, GABRA5, GABRG2, GABRB2, SLC1A2, SLC1A3, NTRK2, CRHR1*) were included in our analysis. SNPs, within these 18 genes, were screened for known genetic associations with suicide, suicidal behaviour and/or associated endophenotypes using biomedical online resources PubMed http://www.ncbi.nlm.nih.gov/pubmed and OMIM http://www.ncbi.nlm.nih.gov/omim. Priority was given to functional SNPs or SNPs within regulatory regions utilising online genetic databases ensembl http://www.ensembl.org/index.html and DbSNP http://www.ncbi.nlm.nih.gov/projects/SNP/.

### Genotyping

DNA was extracted from patient blood samples using standard techniques. Flanking SNP sequences were obtained from DbSNP and all genotyping assays were designed and validated by KBioscience (Hertfordshire, UK). Genotyping for SNP analysis was performed by KBioscience using the competitive allele specific PCR (KASP) chemistry coupled with a FRET-based genotyping system http://www.kbioscience.co.uk/reagents/KASP/KASP.html. Genotyping data was viewed using SNPviewer (KBioscience, Hertfordshire, UK), allowing graphical viewing of the clusters that group the allele calls.

SNPs in linkage disequilibrium (LD) with SNPs of interest were identified using a combination of information from Ensembl http://www.ensembl.org/index.html and Haploview http://www.broadinstitute.org/haploview/haploview.

### Data analysis

Deviations from Hardy Weinberg Equilibrium (HWE) were calculated using the exact test implemented by SNP and Variation Suite (SVS) 7 software (Bozeman, MT 59718 USA; http://www.goldenhelix.com/SNP_Variation/svstrial.html).

Statistical tests of association examining the relationship between patient's alleles or genotype, at each of the 28 genetic loci, and suicide attempt were performed using Pearson's Chi-square test or Fisher's exact test (when counts are low). This analysis was performed both in SPSS and SNP and Variation Suite for verification purposes.

SNPs which showed a modest level of association in the original allelic and genotype tests of association (P < 0.1) and one SNP which showed a gender specific association with SA, were further analysed by applying four genotype models (Additive (XX = 0, XY = 1, YY = 2), Dominant (XX = 0, XY & YY = 1), Over-dominant (XX & YY = 0, XY = 1) and Recessive (XX & XY = 0, YY = 1); X = Major allele, Y = Minor allele). For all models, except the additive model, Pearson's Chi-square test of association was used. For the additive model an exact Armitage trend test was preformed. Binary logistical regression analyses were performed to evaluate the contribution of the genotype model associated with SA at each locus in the prediction of attempted suicide, correcting for potential confounders such as age, gender and certain psychiatric disorders and to interrogate associated SNPs for potential GxG and GxE interactions. Analysis of potential GxE interactions were applied to SNPs with evidence of association in at least one genetic model (n = 3) and to one SNP which showed evidence of association in a gender specific manner (n = 1) and not examined for all candidate SNPs listed in this study. In addition, GXG interactions analysis was performed on these 4 SNPs, which showed evidence of association with SA and not examined for all candidate SNP listed in this study.

A non-parametric Mann-Whitney test was used to calculate the difference in age between cases and controls. For all other comparisons of clinical and demographic variables a Fisher's exact test or Chi-squared test of association was used. All statistical analysis was performed on SPSS (PASW statistics 18, Chicago, Illinois, USA) and genotypic associations verified using SNP and Variation Suite (SVS) 7 software. For all tests, significance was ascribed at *P *< 0.05.

## Results

### Clinical and psychological features of study population

The mean age of the suicide attempter and non-attempter groups was significantly different (34.72 years versus 40.67 years, respectively, (P = 0.017)). The SA group were more likely to have a diagnosis of major depressive disorder (P = 0.022), a positive first degree family history of attempted suicide (P = 0.014), and a history of child abuse (physical or sexual) (P = 0.047). No other demographic or clinical variables were statistically different between the two groups (Table 1).

### Candidate gene selection

In this study, genes considered important in various neurobiological pathways associated with suicide and/or associated disorders or endophenotypes were chosen. Overall, 18 genes were selected for inclusion in our analysis. The majority of genes (n = 8; *COMT, 5-HT2A, 5-HT1A, 5-HTR1B, TPH1, MAO-A, TPH2, DBH*) are important for monoaminergic transmitter systems (norepinephrine, dopamine and serotonin). Key genes of the GABA inhibitory neurotransmitter system (n = 3; *GABRA5, GABRB2, GABRB2*), glutamatergic excitatory neurotransmitter system (n = 2; *SLC1A2, SLC1A3*), HPA axis (n = 1; *CRHR1*), cannabinoid system (n = 1; *CNR1*), cholesterol transport (n = 1; *ABCG1*), neural growth and differentiation (n = 2; *BDNF *&*NTRK2*) were also selected (see Additional File 1). In total, 28 SNPs present within our 18 candidate genes were identified. The majority of SNPs (24/28 SNPs) had previous evidence of association with suicidal behaviour and/or associated disorders or endophenotypes (see Additional File 1).

### Genetic variants and suicide attempts

Results of basic allelic and genotypic tests of association for each SNP and SA are illustrated in Table 2. Basic allelic and genotypic association analyses revealed two significant associations with SA; rs4755404 (Intronic region of *SLC1A2*; P = 0.024: Chi-square test) and rs6296 (Promoter CpG island of *5-HTR1B*; P = 0.047: Chi-square test) loci. In addition a third SNP, rs2269272 (3'untranslated region (UTR)) in *SLC1A3*; P = 0.075: Chi-square test), with a P value < 0.1 was chosen for further analyses. Next, four genotype models (Additive, Dominant, Over-Dominant and Recessive) were tested for association with attempter status at each locus (Table 3).

**Table 2:** Association of candidate markers

Gene Symbol	Allele	RS number	Significance (p)	
			Allelic	Genotypic
**SLC1A3**	C/T	rs2269272	0.562	0.075
**HTR1B**	C/G	rs6296	0.895	0.047
**SLC1A2**	G/C	rs4755404	0.024	0.069
**COMT**	G/A	rs4680	0.123	0.205
**HTR2A**	G/A	rs6313	0.696	0.568
	C/T	rs6311	0.628	0.307
**HTR1A**	C/G	rs6295	0.714	0.907
**TPH1**	G/T	rs1799913	0.136	0.192
	G/T	rs1800532	0.136	0.192
**TPH2**	A/G	rs7305115	0.647	0.559
**DBH**	T/C	rs2519152	0.377	0.761
**CNR1**	T/G	rs6454674	0.948	0.986
	T/C	rs806368	0.695	0.885
	G/A	rs6801844	0.883	0.612
**BDNF**	C/T	rs6265	0.422	0.650
**ABCG1**	A/G	rs1044317	0.261	0.397
	C/G	rs225374	0.631	0.823
	C/G	rs914189	0.862	0.986
**GABRG2**	C/A	rs211014	0.398	0.180
**GABRB2**	A/G	rs1816072	0.270	0.276
	C/T	rs2491397	0.310	0.594
**NTRK2**	A/G	rs1659400	0.453	0.474
	G/A	rs1187272	0.137	0.388
**CRHR1**	A/G	rs110402	0.805	0.763
	T/G	rs242924	0.935	0.463
	C/T	rs7209436	0.852	0.872
**MAOA**	T/G	rs1799835	NA	NA
**GABRA5**	A/G	rs140681	NA	NA

P value obtained from Pearson's Chi-square test or Fisher's exact test. NA = not applicable, as genotype are identical for case and controls.

**Table 3:** Genotype frequencies and genetic models of association

			Genotype Frequencies			Significance (p value)		
Gene symbol	RS number	Genotype	Controls	SA cases	Additive	Dominant	Over-Dominant	Recessive
**SLC1A3**	rs2269272	CC/CT/TT	0.687/0.229/0.084	0.667/0.32/0.013	0.56	0.787	0.199	**0.042**
**HTR1B**	rs6296	GG/CG/CC	0.59/0.265/0.145	0.5/0.432/0.068	0.847	0.256	**0.028**	0.121
**SLC1A2**	rs4755404	CC/CG/GG	0.451/0.427/0.122	0.28/0.52/0.20	**0.029**	**0.026**	0.243	0.182

P value obtained from Pearson's Chi-square test for all genotype tests, with the exception of the additive model, where an exact Armitage trend test was used. Numbers highlighted in **bold **represent genotype models that were significantly associated with Suicide attempt status (P = < 0.05). SA: Suicide Attempt.

Rs4755404 yielded evidence of association in an additive and dominant genotype model (P = 0.029: Exact Armitage Trends test; P = 0.026: Chi-square test, respectively), where patients with a G/C or G/G genotype were significantly more likely to be present in the SA group. Variation at the rs6296 locus was associated with SA in an over-dominant genotype model, whereby a heterozygote (C/G) genotype was more frequently present in patients in the SA group than non-attempter group (P = 0.028). Rs2269272 variants revealed evidence of association in a recessive genotype model (P = 0.042; Chi-square test), with the T/T genotype more frequent in the non-attempter group than attempter group (Table 3).

SNPs of interest were investigated for possible associations with other clinical variables illustrated in Table 1. SNP rs2269272 showed no evidence of association with other clinical variables in the study. A dominant genotype at the rs4755404 locus was associated with patients with a history of schizophrenia and other psychotic disorders (P = 0.012; Chi-square test). A recessive genotype at the rs6296 locus yielded an association with individuals who abuse/depend on alcohol (p = 0.035). Binary logistical regression analyses were performed to evaluate the contribution of individual risk/protective polymorphisms in the prediction of attempted suicide. After controlling for possible confounders such as age, gender, alcohol abuse/dependence (rs6296 only) and schizophrenia (rs4755404 only), rs2269272 recessive genotype model was no longer a significant predictor of SA (P0.121; Odds ratio (OR) = 0.83, 95% C.I: .022-1.563). Rs4755404 dominant genotype model and rs6296 over-dominant genotype model were still significant predictors of SA (P = 0.031; OR = 2.198; 95% CI 1.059-4.563 and P = 0.023; OR = 2.244; 95% CI 1.096-4.597, respectively).

Each locus was also analysed for deviations from HWE. Deviations from HWE were observed at the rs6296 locus in the non-attempter group (P = 0.004) and at rs2269272 locus in the non-attempter group (P = 0.014).

### Genetic and environmental interaction analysis

Individual SNPs were analysed for evidence of gene by sex interaction, to identify possible sex-specific associations with SA. Two gene-sex interactions were observed. A sex by rs2269272 interaction was found where a T/T genotype was more frequent in non-attempter male SA group. A gene by sex interaction was also observed for an intronic SNP, rs1659400, in the *NTRK2 *gene. Controlling for age, a C/C genotype at this locus was a significant predictor of SA in females (P = 0.048; OR = 2.968; 95% C.I 1.01- 8.72).

Finally, we investigated the effect of susceptibility loci on suicide attempts by other susceptibility loci by examining various two and three way G×G interactions. Logistic regression was used to model SA risk as a function of the genotype models associated with SA at each locus and 2-locus and 3-locus G×G interactions (Table 4). Controlling for possible confounders, age, gender, alcohol abuse/dependence and schizophrenia, a 3-locus gene (G×G×G) interaction (rs6296 (C/G) × rs4755404 (C/G or G/G) × rs1659400 (C/C) was a significant predictor of SA (P = 0.046).

**Table 4:** Final gene by gene interaction model: Logistic regression analysis

							95% C.I for OR	
**Variable**	**B**	**S.E**	**Wald**	**df**	**P value**	**OR**	**Lower**	**Upper**

Age	-.043	.015	8.305	1	.004	.958	.930	.986
Sex	-1.062	.422	6.349	1	.012	.346	.151	.790
alcohol abuse/dependence	1.164	.422	7.598	1	.006	3.204	1.400	7.333
schizophrenia and other psychotic disorders	- 0.139	.810	.029	1	.864	.870	.178	4.257
rs6296	1.104	.868	1.615	1	.204	3.015	.550	16.539
rs4755404	1.291	.593	4.736	1	.030	3.636	1.137	11.628
rs1659400	.953	.934	1.041	1	.308	2.592	.416	16.159
rs6296 by rs4755404	-1.141	1.065	1.150	1	.284	.319	.040	2.573
rs6296 by rs1659400	-1.356	1.440	.887	1	.346	.258	.015	4.332
rs1659400 by rs4755404	-2.060	1.139	3.275	1	.070	.127	.014	1.187
rs6296 by rs4755404 by rs1659400	3.613	1.811	3.982	1	.046	37.087	1.067	1289.592
Constant	.592	.778	.579	1	.447	1.808		

B, unstandardised logistic regression coefficient; S.E, Standard error; Wald, Wald statistic; df, Degrees of freedom; OR, Odds ratio; C.I., Confidence interval

To evaluate further the association between susceptibility genotypes only and environmental risk factors, logistic regression was used to model risk as a function of the genotype models associated with SA at each locus, environmental risk factors and their interaction. Results provide evidence of a possible G (C/C genotype) × E (history of childhood abuse (both physical and sexual)) interaction at the rs1659400 locus. Interestingly, rs1659400 was not associated with SA (p = 0.823, Chi-square), however when all variables (including gender and age) are entered into a logistic regression analysis, the G×E interaction term is close to significance (P = 0.056, Table 5). Moreover, since a C/C genotype was associated with female SA, a G×E interaction was examined in females only. A G×E interaction contributed to the predictive ability of the model (P = 0.054, Table 5), a trend which did not reach statistical significance. Taken together our results suggest that individuals who experienced childhood abuse (physical or sexual) and have a C/C genotype at the rs1659400 locus may be more susceptible to SA.

**Table 5:** Gene by environment interaction: Logistic regression analysis

							95% C.I for OR	
**Variable**	**B**	**S.E**	**Wald**	**df**	**P value**	**OR**	**Lower**	**Upper**

Sex	-.384	.358	1.152	1	.283	.681	.338	1.373
Age	-.035	.013	7.669	1	.006	.965	.941	.990
rs1659400	-.038	.426	.008	1	.928	.962	.417	2.219
abuse	-.508	.469	1.175	1	.278	.602	.240	1.508
rs1659400 by abuse	1.518	.795	3.641	1	.056	4.562	.960	21.684
Constant	1.406	.627	5.035	1	.025	4.079		
***Females Only***								
Age	-.025	.018	1.911	1	.167	.975	.941	1.010
rs1659400	.166	.634	.068	1	.794	1.181	.341	4.093
abuse	.003	.774	.000	1	.997	1.003	.220	4.571
rs1659400 by abuse	2.653	1.378	3.707	1	.054	14.202	.953	211.572
Constant	.633	.848	.556	1	.456	1.883		

B, unstandardised logistic regression coefficient; S.E, Standard Error; Wald, Wald statistic; df, degrees of freedom; OR, Odds ratio; C.I., Confidence interval

## Discussion

We have identified 4 SNPs (rs4755404, rs2269272, rs6296 and rs1659400) in genes *SLC1A2, SLC1A3, 5-HTR1B *and *NTRK2*, respectively, which showed evidence of association with SA compared to a non-attempter psychiatric control group. At present, there is no published data on 3/4 of the genetic variants (rs4755404, rs2269272 and rs1659400) reported here and their association with suicide or SA. In addition, we identified a 3-locus (rs6296 (C/G) × rs4755404 (C/G or G/G) × rs1659400 (C/C)) G×G interaction, which was a significant predictor of SA, suggesting that variation in *SLC1A2, 5-HTR1B *and *NTRK2 *genes may contribute to the risk of SA independently, and in an interactive manner. This paper provides evidence for the first time of a putative G×E interaction, where genetic variation at the rs1659400 locus may moderate the risk associated with history of childhood abuse and subsequent suicidal acts.

*SLC1A2 *and *SLC1A3 *are glial high-affinity transporter molecules that regulate glutamate concentrations at synapses [15]. Aberrant expression of these key transporters could impair reuptake of glutamate, hence prolong synaptic activation and potentially lead to cytotoxic damage to neurons and glia [15]. *SLC1A2 *and *SLC1A3 *are downregulated in the brains of MDD patients compared to controls [28]. Moreover, decreased *SLC1A3 *expression has been observed in suicide brains [29].

In this study, the *SLC1A2 *gene variant, rs4755404, was associated with SA and schizophrenia and other psychotic disorders. Consistent with these findings, rs4755404 was previously associated with schizophrenia in a Japanese patient cohort [30]. In contrast, the *SLC1A3 *gene variant, rs2269272, was significantly associated with male non-attempters, where a gene-sex interaction was observed. The T/T genotype is absent from the attempter group and over-represented in the male non-attempter group, suggesting a T/T genotype may confer a protective effect on risk of SA, particularly in males with underlying psychiatric disorders. HWE analysis revealed that the control group was not in equilibrium for the rs2269272 locus, which is likely due to the fact that our control group is not "disease-free" but rather consists of individuals with underlying psychiatric conditions. However, the possibility that some other factor may be influencing the over-representation of T allele homozygotes in the non-attempter group cannot be ruled out. Therefore, further investigation in a larger cohort of patients is warranted. Taken together, our findings support the glial hypothesis of mood abnormalities [28] and concur with the literature on a putative role of glutamate dysregulation in suicidal behaviour.

The serotonin (5-HT) system is the most widely studied area of neurobiological suicide research [21]. Aberrant 5-HT receptor binding has been implicated in suicide and SA [12]. *5-HTR1B *binding is decreased in the prefrontal cortex of suicide brains [31]. Previously a *5-HTR1B *promoter CpG island genetic variant, rs6296, was associated with SA [32]. However, findings are inconsistent and a number of studies have found no evidence of association [33,34]. Rs6296 has also been associated with substance use disorders, major depression and inconsistently with alcohol abuse [33,35]. Here we report a significant association between the rs6296 locus and SA. In addition, persons with a C/G genotype and a history of abuse or dependence on alcohol were significantly associated with SA, but a gene × alcohol interaction was not evident, suggesting that they represent additive risk factors. These findings provide the impetus for future studies in a cohort of alcohol abuse/dependence patients to further understand the relevance of rs6296 genetic variants and SA in patients with a history of alcohol abuse/dependence.

*NTRK2 *(*TRKB*) encodes the receptor for BDNF. Aberrant neurotrophic signalling has been implicated in suicide risk by various studies [36,37]. *BDNF *and *NTRK2 *mRNA and protein expression are reported downregulated in the prefrontal cortex and hippocampus of suicide victims compared to controls [36,37]. The majority of studies have focused on functional *BDNF *variant, rs6265, which has been inconsistently implicated in suicide and MDD [38,39]. In this study, we found no evidence of association between rs6265 and SA, consistent with a previous report [40]. Previously, a number of genetic variants within the *NTRK2 *gene have been associated with SA among depressed patients [41]. We observed a significant association with *NTRK2 *intronic genetic variant, rs1659404, and SA in females. Rs1659400 is in strong LD with several other SNPs within the *NTRK1 *gene, some of which have been implicated in alcohol dependence and depression [42,43].

To date a number of gene × childhood adversity interactions have been reported in psychiatric patients [44-46]. Childhood trauma (including abuse) has been reported to interact with low expressing *5-HTTLPR *genotypes and moderate the risk of suicidal behaviour [47]. Recently, an interaction between child maltreatment and *5-HTT *polymorphisms and suicidal ideation among children was described [48]. Here we report, for the first time, a possible moderation effect of a *NTRK2 *polymorphism on childhood abuse and risk for future suicidal acts. It is important to note that a history of childhood abuse was assessed in this study by a trained clinician as opposed to self-report questionnaires. Recent research has demonstrated that clinician ratings of developmental histories, such as childhood sexual and physical abuse, are in agreement with patient's self-reports and thus supports the validity of clinician reports for numerous clinical variables, including childhood abuse assessment [49]. Future studies could investigate the putative interaction of this genetic variant with childhood trauma score, which would include sexual, physical and emotional abuse and neglect. Such a study would provide a more comprehensive assessment of gene × childhood adversity interaction and risk for SA at this locus.

A number of limitations are apparent in the current study. Firstly, case/control samples were not age-matched, with the SA group having a significantly younger mean age. The SA group also contained a greater number of individuals with a family history of SA and a history of childhood abuse. In addition, rates of MDD diagnoses were different in SA and non-attempter psychiatric controls. However, the genetic variants identified in this study were not associated with either MDD, a family history of SA or abuse. Moreover, our sample size may have reduced power to detect small genetic effects, or alternative interactions between genetic and environmental risk factors. In this study, multiple testing correction, such as Bonferroni correction, was not applied. Bonferroni correction can be regarded as ultraconservative [50] and ignores the functional candidate gene study design utilised, which is likely to increase the prior probability of detecting an association. In addition, given our modest sample size, Bonferroni correction is likely to result in large type II error rates. Arguably the best solution to type I error is replication [50].

## Conclusion

In conclusion, we have identified statistically as well as biologically significant genetic polymorphisms that may be relevant to the underlying diathesis for suicidal acts [1]. An understanding of the functional consequence of these SNPs from a neuropathological standpoint would be advantageous to advance our understanding of their role in SA. The SNP associations identified in this study, although modest, merit further investigation in a larger cohort of suicide attempters and may contribute to future meta-analysis of suicidal behaviour susceptibility loci. Moreover, follow-up research of the genetic susceptibly polymorphisms identified in this study may increase our understanding of the complex relationship of genetic and environmental factors in suicidal behaviour in major psychiatric disorders.

## List of abbreviations

DZ: Dizygotic; GxG: Gene-Gene; GABA: Gamma Aminobutyric Acid; GxE: Gene-Environment; HPA: Hypothalamic-Pituitary-Adrenal; HWE: Hardy Weinberg Equilibrium; INSURE: Ireland North/South Urban/Rural Epidemiologic; LD: Linkage Disequilibrium; MZ: Monozygotic; OR: Odds Ratio; SA: Suicide Attempts; SNPs: Single Nucleotide Polymorphisms; SVS: SNP and Variation Suite; UTR: Untranslated Region

## Contributions of Authors

TMM performed genetic and statistical analysis, interpretation of the data and drafted the manuscript. MR carried out blood collection and Clinical rating and contributed to study design. TF participated in the design of the study and helped to draft the manuscript. CK participated in the design of the study. RM participated in the design of the study. JO'G carried out blood collection and Clinical rating. EC participated in the design of the study. JB carried out blood collection and Clinical rating. MR carried out blood collection and Clinical rating. AJ participated in the design of the study. AM carried out blood collection and Clinical rating. NB carried out blood collection and Clinical rating. AC carried out blood collection and Clinical rating DL carried out Clinical rating. SB carried out Clinical rating. PT participated in statistical and genetic analysis and helped to draft the manuscript. AB extracted DNA and prepared sample for storage and genotyping. LQ participated in statistical analysis. LD participated in statistical and genetic analysis and helped to draft the manuscript. CK participated in the design of the study and help to draft the manuscript. KMM participated in the design of the study and help to draft the manuscript. All authors have read and approved the final manuscript.

## Competing interests

The authors declare that they have no competing interests.

## Supplementary Material

Additional file 1 **Summary of candidate genes**. A comprehensive list of all gene names, symbols and SNPs studied in the current report Click here for file
